# Clinical mutational profiling of 1006 lung cancers by next generation sequencing

**DOI:** 10.18632/oncotarget.18042

**Published:** 2017-05-20

**Authors:** Peter B. Illei, Deborah Belchis, Li-Hui Tseng, Doreen Nguyen, Federico De Marchi, Lisa Haley, Stacy Riel, Katie Beierl, Gang Zheng, Julie R. Brahmer, Frederic B. Askin, Christopher D. Gocke, James R. Eshleman, Patrick M. Forde, Ming-Tseh Lin

**Affiliations:** ^1^ Department of Pathology, Johns Hopkins University School of Medicine, Johns Hopkins Hospital, Baltimore, Maryland, USA; ^2^ Department of Medical Genetics, National Taiwan University Hospital, Taipei, Taiwan; ^3^ Division of Hematology and Bone Marrow Transplantation, University of Udine Hospital, Udine, Italy; ^4^ Department of Oncology, Johns Hopkins University School of Medicine, Johns Hopkins Hospital, Baltimore, Maryland, USA

**Keywords:** lung, mutation, sequencing, cancer, profiling

## Abstract

Analysis of lung adenocarcinomas for actionable mutations has become standard of care. Here, we report our experience using next generation sequencing (NGS) to examine *AKT1*, *BRAF*, *EGFR*, *ERBB2*, *KRAS*, *NRAS*, and *PIK3CA* genes in 1006 non-small cell lung cancers in a clinical diagnostic setting. NGS demonstrated high sensitivity. Among 760 mutations detected, the variant allele frequency (VAF) was 2–5% in 33 (4.3%) mutations and 2–10% in 101 (13%) mutations. A single bioinformatics pipeline using Torrent Variant Caller, however, missed a variety of *EGFR* mutations. Mutations were detected in *KRAS* (36% of tumors), *EGFR* (19%) including 8 (0.8%) within the extracellular domain (4 at codons 108 and 4 at codon 289), *BRAF* (6.3%), and *PIK3CA* (3.7%). With a broader reportable range, exon 19 deletion and p.L858R accounted for only 36% and 26% of *EGFR* mutations and p.V600E accounted for only 24% of *BRAF* mutations. NGS provided accurate sequencing of complex mutations seen in 19% of *EGFR* exon 19 deletion mutations. Doublet (compound) *EGFR* mutations were observed in 29 (16%) of 187 *EGFR*-mutated tumors, including 69% with two non-p.L858R missense mutations and 24% with p.L858 and non-p.L858R missense mutations. Concordant VAFs suggests doublet *EGFR* mutations were present in a dominant clone and cooperated in oncogenesis. Mutants with predicted impaired kinase, observed in 25% of *BRAF*-mutated tumors, were associated with a higher incidence of concomitant activating *KRAS* mutations. NGS demonstrates high analytic sensitivity, broad reportable range, quantitative VAF measurement, single molecule sequencing to resolve complex deletion mutations, and simultaneous detection of concomitant mutations.

## INTRODUCTION

Approximately 30–40% of Asian patients and 10–15% of Caucasian patients with lung adenocarcinoma harbor activating mutations in the *epidermal growth factor receptor* (*EGFR*) gene. Gefitinib, erlotinib and afatinib are tyrosine kinase inhibitors (TKI) approved by the Food and Drug Administration (FDA) of the United States for treatment of patients with *EGFR*-mutated lung cancers [1–3]. In 2011, a provisional clinical opinion from the American Society of Clinical Oncology recommended testing for *EGFR* mutations in patients with metastatic lung cancer to predict response to TKI therapy [4]. Molecular testing guidelines for selection of lung cancer patients for TKI therapy have been published and are currently under revision by the College of American Pathologists, International Association for the Study of Lung Cancer, and Association for Molecular Pathology [5].

A variety of molecular diagnostic assays have been clinically validated for detection of *EGFR* mutations [6]. Although the prior gold standard of Sanger sequencing covers all *EGFR* mutations within exons 18–21, it’s analytic sensitivity (20–40% tumor cellularity) may not be adequate in the clinical diagnostic setting where specimens containing low tumor cellularity are not uncommon [7, 8]. The analytic sensitivity can be improved to approximately 5% variant allele frequency (VAF) (10% tumor cellularity) using pyrosequencing, 1% VAF using mutation-specific real time PCR assays, or even less than 1% by droplet digital PCR. Currently, there are two assays approved by the FDA for testing *EGFR* mutations in lung cancers, the cobas EGFR mutation test (Roche Molecular Systems, Branchburg, NJ) and the therascreen EGFR RGQ PCR Kit (Qiagen, Hilden, Germany) [9–13]. Both assays detect hot spot *EGFR* mutations by multiple separate runs of mutation-specific real-time PCR assays. These assays are not able to detect less common mutations outside the reportable ranges. A total of 150 ng DNA is needed for the cobas test. DNA input has not been quantified for the therascreen test. We have shown that 44% of specimens submitted for clinical mutational profiling were taken by biopsy or fine needle aspiration [14]. DNA isolated from biopsy or fine needle aspiration specimens containing limited tissue may not be sufficient.

Multiplexed genotyping platforms are replacing the traditional “one test-one drug” paradigm not only because of continuous expansion of predictive markers for targeted therapeutics but also often limited tissues submitted to the clinical diagnostic laboratories. Primer extension-based assays with a multiplex design, such as the Sequenom MassARRAY system, detect multiple hotspots within a panel of genes including *EGFR* in a single reaction while retaining an analytic sensitivity of 5% or less VAF [15, 16]. In the era of precision cancer medicine, molecular diagnostics assays with a higher analytic sensitivity and a broader reportable range are warranted to provide a more comprehensive mutational profiling. Next generation sequencing (NGS) technology has led to a revolution in genome discovery and will soon become the most cost-effective multiplexed sequencing platform in the setting of clinical care [7, 17]. We have previously validated a NGS platform using the AmpliSeq Cancer Hotspot Panel and Personal Genome Machine in a *Clinical Laboratory Improvement Amendments* (CLIA)-certified laboratory [18]. In this retrospective analysis for quality assessment, we surveyed our experience with clinical mutation detection of *AKT1*, *BRAF*, *ERBB2*, *EGFR*, *KRAS*, *NRAS*, and *PIK3CA* genes in 1006 lung cancer specimens using this NGS assay, including false negative calling, the capability of detecting complex deletion mutations within exon 19 of the *EGFR* gene, a high frequency of doublet (compound) *EGFR* mutations with concordant VAFs, and the association of kinase impaired *BRAF* mutations with activating *RAS* mutations.

## RESULTS

### Positive and negative controls

No mutations were detected in 88 runs of the negative control specimen while all mutations in the positive control specimens were detected. The observed VAFs in the positive controls were highly consistent throughout the test period, demonstrating that NGS is a precise quantification assay for VAF (Supplementary Table 1).

### Mutations missed by Torrent Variant Caller

**Figure 1:**
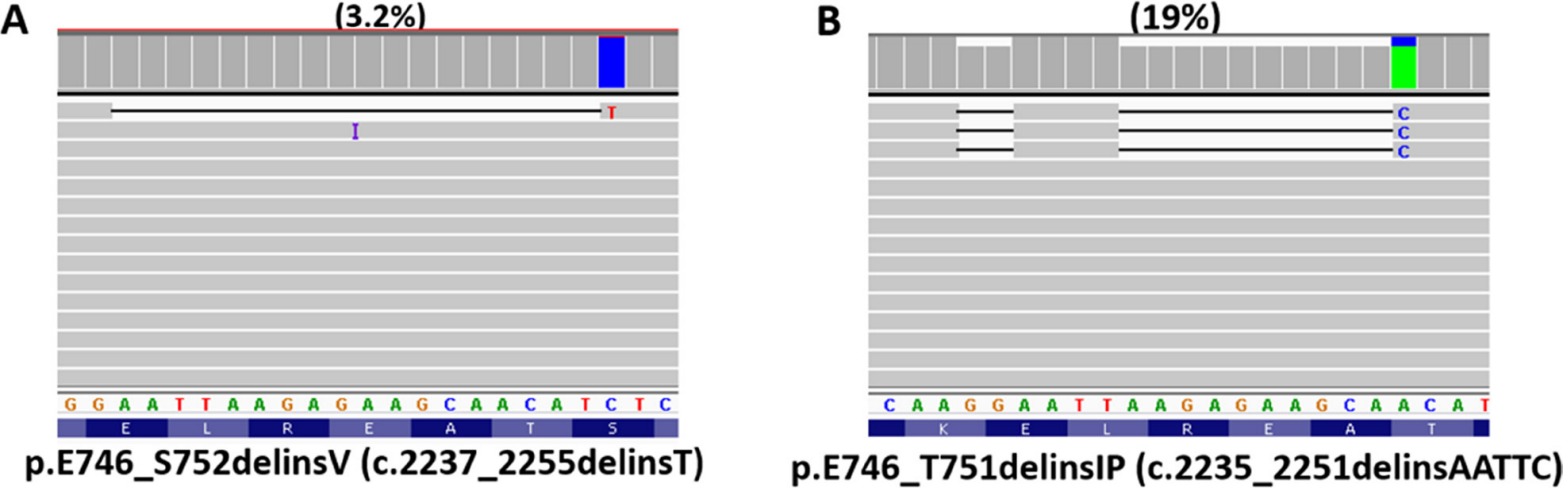
*EGFR* mutations detected by next generation sequencing *EGFR* exon 19 deletion mutation of 3.2% variant allele frequency detected by Integrative Genomics Viewer inspection, but missed by Torrent Variant Caller (**A**). Complex exon 19 deletion mutation composed of two deletions (c.2235_2236del and c.2241_2250del) and one single nucleotide change (c.2251A>C) (**B**).

Specimens with prior TKI therapy were not included in this study. One mutation was detected in 560 of 1006 tumors, 2 mutations in 70 tumors, and 3 mutations in 3 tumors. Our analysis pipeline included both Torrent Variant Caller and direct visual inspection of all amplicons within the reportable range using Integrative Genomics Viewer (IGV). A total of 15 mutations detected by IGV inspection were missed by Torrent Variant Caller (Table 1). These included 12 *EGFR* mutations (3 missense mutations of exon 18, 3 deletion mutations of exon 19, 3 insertion/duplication mutations of exon 20 and 3 p.L858R mutations of exon 21), 2 *PIK3CA* missense mutations and one *ERBB2* duplication mutation. VAFs ranged from 3.2% to 65% (Figure 1A). Eleven of the 15 false negative calls by Torrent Variant Caller occurred within the first year. The false negative calls were most likely related to bioinformatics pipelines of the Torrent Variant Caller, which has been improved with the updated versions. The recent Torrent Variant Caller did not miss mutations in lung cancers. All insertion/deletion (indel) mutations detected within *EGFR* exons 19 and 20 by a capillary electrophoresis sizing assay were also detected by IGV inspection. Accordingly, sizing by capillary electrophoresis was discontinued in April 2014.

**Table 1:** Mutations detected by integrative genomics viewer inspection but missed by Torrent Variant Caller (VC)

Gene^a^	cDNA change	Amino acid change	VAF^b^	VC version^c^
*EGFR* (Re)	c.2573T>G	p.L858R	8.1	3.4.51874
*EGFR* (Re)	c.2171G>C	p.G724A	16	3.4.51874
*PIK3CA* (Bx)	c.1634A>C	p.E545A	10	3.4.51874
*EGFR* (FNA)	c.2237_2255delinsT^d^	p.E746_S752delinsV	3.2	3.4.51874
*EGFR* (Bx)	c.2156G>C	p.G719A	8.9	3.4.51874
*EGFR* (Re)	c.2573T>G	p.L858R	6.1	3.4.51874
*EGFR* (Re)	c.2573T>G	p.L858R	12	3.4.51874
*EGFR* (FNA)	c.2236_2250del	p.E746_A750del	5.5	3.6.63335
*EGFR* (Bx)	c.2300_2308dup	p.A767_V769dup	9.4	3.6.63335
*EGFR* (Bx)	c.2302_2303insCGCTGGCCA	p.A767_S768insTLA	5	3.6.63335
*EGFR* (Re)	c.2156G>C	p.G719A	32	3.6.63335
*ERBB2* (FNA)	c.2313_2324dup	p.A771_M774dup	7.6	3.6.63335
*EGFR* (Bx)	c.2311_2312delinsGGGTT^e^	p.N771delinsGF	65	3.6.63335
*PIK3CA* (Bx)	c.1193G>T	p.R398L	5.5	3.6.63335
*EGFR* (Re)	c.2239_2256del	p.L747_S752del	36	3.6.63335

^a^Re: resection specimens, Bx: biopsy specimens; FNA: fine needle aspiration specimens. Two specimens (*EGFR* p.N771delinsGF mutation and *PIK3CA* p.R398L mutation) were concentrated by using the Amicon filter.

^b^VAF: variant allelic frequency. Depth of coverage was more than 1000 reads except the specimen with *PIK3CA* p.R398 mutation (450 reads).

^c^The current VC version (5.0.2.1) did not miss mutations detected by IGV since December 2015.

^d^The c.2237_2255delinsT mutation was composed of c.2237_2254del and c.2255C>T. Both were missed by Torrent Variant Caller.

^e^The c.2311_2312delinsGGGTT mutation was composed of c.2310-2311insGGG, c.2311A>T and c.2312A>T. The Torrent Variant Caller detected c.2311A>T and c.2312A>T, but not c.2310-2311insGGG.

### *EGFR* mutations

A total of 49 unique *EGFR* variants/mutations were detected in 187 (19%) tumors, including 29 tumors with 2 *EGFR* mutations and 10 variants/mutations outside exons 18–21 involving extracellular domain of EGFR (four codon 108 mutations, four codon 289 mutations and two possible germline variants, p.E282K and p.H584R) (Table 2 and Supplementary Table 2). All except p.L858fs*45 caused either missense mutations or in-frame indel mutations. Distribution of the 216 *EGFR* mutations was shown in Supplementary Figure 1 and frequencies of TKI sensitive or resistant mutations among the 187 *EGFR-*mutated tumors were shown in Supplementary Figure 2.

**Table 2:** Mutational profiling of 1006 lung cancers and variant allele frequency

	Lung cancer	Variant allele frequency^a^		
	(*n* = 1006)	≤ 5%	≤ 10%	≤ 20%
*AKT1*	4 (0.4%)	NA	NA	NA
*BRAF*	63 (6.3%)	7.7%	25% (*P* = 0.009)	63% (*P* < 0.001)
*EGFR*	187 (19%)	2.2%	7.8% (*P* = 0.07)	27% (*P* = 0.003)
*ERBB2*	13 (1.3%)	NA	NA	NA
*KRAS*	362 (36%)	4.1%	13%	39%
*NRAS*	11 (1.1%)	NA	NA	NA
*PIK3CA*	37 (3.7%)	13%	37% (*P* < 0.001)	63% (*P* = 0.003)

NA: not analyzed.

^a^Percentage of mutations with variant allele frequencies of ≤ 5%, ≤ 10% and ≤ 20%. Data of all 1074 specimens were included for analysis of variant allele frequencies. *P* values were analyzed by comparing with *KRAS* variant allele frequencies.

At the level of cDNA, there were 5 unique simple exon 19 deletion mutations (defined as an exon 19 mutation with no additional nucleotide changes within 1–2 adjacent codons) and 11 unique complex exon 19 deletion mutations (defined as an exon 19 deletion accompanied with one or more nucleotide changes in *cis* position within 1–2 adjacent codons) (Table 3). Complex mutations were seen in 15 (19%) of 78 exon 19 deletion mutations, including 12 with one deletion and 1 or 2 single nucleotide changes, 2 with two deletions, and one with two deletions and one single nucleotide change (Figure 1B). All the accompanied single nucleotide changes were located 3’ to the deletion. All 5 simple deletion mutations were observed in 2 or more tumors while most complex deletion mutations were observed in one tumor.

**Table 3:** Simple and complex exon 19 deletion mutations of the *EGFR* gene

	cDNA change	Amino acid change	Number of cases
Simple deletion			
	c.2235_2249del	p.E746_A750del	35
	c.2236_2250del	p.E746_A750del	16
	c.2240_2254del	p.L747_T751del	3
	c.2239_2256del	p.L747_S752del	2
	c.2240_2257del	p.L747_P753delinsS	7
Complex deletion			
c.2236_2248delinsGCAC (c.2236_2244del, c.2246A>C, c.2248G>C)^a,b^		p.E746_A750delinsAP	1
c.2235_2251delinsAATTC (c.2235_2236del, c.2241_2250del, c.2251A>C)^b^		p.E746_T751delinsIP	1
c.2237_2252delinsT (c.2237_2251del, c.2252C>T)^b^		p.E746_T751delinsV	1
c.2237_2255delinsT (c.2237_2254del, c.2255C>T)		p.E746_S752delinsV	2
c.2237_2257delinsTCT (c.2237_2253del, c.2257del)^b^		p.E746_P753delinsVS	1
c.2239_2248delinsC (c.2236_2244del, c.2248G>C)		p.L747_A750delinsP	3
c.2238_2248delinsGC (c.2236_2244del, c.2247A>G, c.2248G>C)		p.L747_A750delinsP	1
c.2239_2251delinsC (c.2238_2249del, c.2251A>C)		p.L747_T751delinsP	2
c.2239_2252delinsCA (c.2239_2248del, c.2251_2252del)^a,b^		p.L747_T751delinsQ	1
c.2239_2258delinsCA (c.2239_2256del, c.2258C>A)		p.L747_P753delinsQ	1
c.2240_2264>CGAAAGG (c.2240_2257del, c.2264C>G)^a,b^		p.L747_A755delinsSKG	1

^a^Undetectable or detectable with a lower analytic sensitivity by cobas EGFR test.

^b^Undetectable or detectable with a lower analytic sensitivity by therascreen EGFR test.

### Mutations in the mitogen-activated protein kinase (MAPK) pathway

A total of 19 unique *KRAS* mutations were detected in 362 (36%) tumors, including 3 tumors with two *KRAS* mutations (Table 2 and Supplementary Table 3). These mutations were located within exon 2 in 326 (89%) of 365 *KRAS* mutations, exon 3 in 9.0% mutations, and exon 4 in 1.6% mutations. The two most common *KRAS* mutations were p.G12C (34%) and p.G12V (22%). Mutations located outside codon 12 or 13 were observed in 43 tumors (12% of *KRAS*-mutated tumors). Six unique *NRAS* mutations were detected in 11 tumors (1.1%), 6 at codon 12, 4 at codon 61 and one p.G48A (Table 2 and Supplementary Table 3).

Twenty-four unique *BRAF* mutations were detected in 63 (6.3%) tumors (Table 2 and Supplementary Table 4). The most common residue involved by *BRAF* mutations was codon 600 (25%), followed by codon 469 (18%), codon 466 (16%), codon 594 (14%), and codon 601 (11%) (Supplementary Figure 3). The p.V600E mutant accounted for only 24% of *BRAF* mutations and 40% of *BRAF* mutations occurred within exon 11. Of the 63 *BRAF*-mutated tumors, BRAF kinase activity was predicted to be elevated in 33 (52%), impaired in 16 (25%) and unknown in 14 (22%) tumors. The most common kinase-impaired *BRAF* mutants are p.G466V (5) and p.D594G (5), followed by p.D594N (3), p.G466E (1), p.G466R (1) and p.D594H (1).

### Mutations in the phosphatidylinositol 3-kinase (mTOR) pathway

*AKT1* mutations were detected in 3 tumors with p.E17K and one with p.R25H (Table 2 and Supplementary Table 5). Seventeen unique *PIK3CA* mutations were detected in 37 (3.7%) tumors (Table 2 and Supplementary Table 5). All except p.R401* were missense mutations. Most mutations were located within exons 9 (57%), 20 (22%) and 4 (11%). The 3 most common codons (E542, E545 and H1047) accounted for only 65% of the *PIK3CA* mutations.

### *ERBB2* mutations

Five unique *ERBB2* mutations were detected in 13 (1.3%) tumors (Table 2 and Supplemental Table 5). There were 12 insertion/duplication mutations within exon 20 and one missense mutation within exon 19. The most common mutation was p.A771_M774dup (62% of *ERBB2* mutations).

### Variant allele frequency

A total of 760 mutations were detected in the 1074 specimens. VAFs were 2–5% in 33 (4.3%) mutations, 2–10% in 101 (13%) mutations, and 2–20% in 289 (38%) mutations (Table 2). VAFs were 5% or less in 2.2% of *EGFR* mutations and 10% or less in 7.8% of *EGFR* mutations. VAFs of the *KRAS* gene (27% ± 18%, mean ± standard deviation, *P* < 0.001 by Student’s *t*-test) were significantly lower than those of the *EGFR* gene (35% ± 20%) and significantly higher than those of the *BRAF* (20% ± 16%, *P* < 0.001) and *PIK3CA* genes (20% ± 19%, *P* = 0.005). The proportions of *BRAF*-mutated specimens and *PIK3CA*-mutated specimens with < 10% or < 20% VAFs were significantly higher than those of *KRAS*-mutated specimens and *EGFR*-mutated specimens (Table 2).

### Doublet (compound) *EGFR* mutations

**Figure 2:**
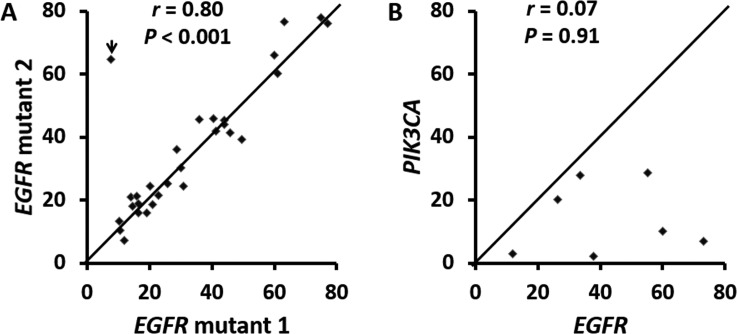
Correlation of variant allele frequencies in 29 tumors with doublet (compound) *EGFR* mutations (**A**) and 7 tumors with concomitant *EGFR* and *PIK3CA* mutations (**B**). The *r* is 0.94 when an outlier (arrowhead in A) was removed. This outlier represents a tumor with a p.T790M germline mutation and a p.L858 somatic mutation. *r*: Spearman’s rank correlation coefficiency.

**Figure 3:**
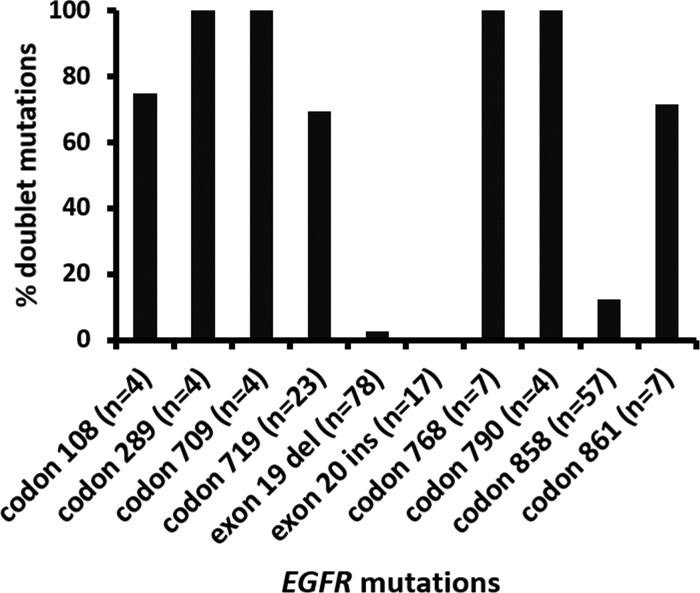
Incidence of doublet (compound) *EGFR* mutations involving different codons Exon 19 del: exon 19 deletion mutations. Exon 20 ins: exon 20 insertion/duplication mutations.

There were 29 tumors with two *EGFR* mutations including 20 (69%) tumors with two non-p.L858R missense mutations, 7 (24%) with p.L858R and non-p.L858R missense mutation, one with p.L747_P753delinsS and p.T790M and one with p.L747_T751del and p.K754Q (Table 4). VAFs of doublet mutations were highly concordant (Figure 2A, *r* = 0.80) except for a tumor with 7.6% p.L858R and 65% p.T790M in a context of 11–30% estimated tumor cellularity, suggesting a germline p.T790M mutation. Doublet mutations were uncommon in tumors with exon 19 deletion (2 of 78, 2.6%) and exon 20 insertion (0 of 18) (Figure 3). Among the single nucleotide mutations, p.L858R (12%) showed a significantly lower incidence of concomitant *EGFR* mutations than other single nucleotide mutations, 100% in codons 289, 709, 768 and 790 (all *P* values < 0.001) and 70% to 75% in codons 108 (*P* = 0.01), 719 (*P* < 0.001) and 861 (*P* = 0.002) (Figure 3). These included 6 tumors with p.S768I and p.G719A/C/S, one with p.S768I and p.L858R and 4 with the resistance mutation, p.T790M (two with p.L858R, one with p.G719A, and one with exon 19 deletion). There were no coexisting mutations between codon 719 mutations, exon 19 deletion, exon 20 insertion, and p.L858R. All the partners of codon 719 mutations were those less common missense mutations, including 3 tumors with codon 709 mutations of the same alleles. Doublet mutations were observed in 7 of 8 tumors with mutations involving the extracellular domain (codons 108 and 289) including 3 tumors with p.L858R, two with codon 724 mutation, one with p.L861Q, and one with p.G719C.

**Table 4:** Doublet (compound) *EGFR* mutations in 1006 lung cancers

mut 1^a^	Exon (mut 1)	mut 2^a^	Exon (mut 2)
p.G724A (17%)	18	p.R108G (16%)	3
p.G719C (44%)	18	p.A289V (45%)	7
p.G724S (50%)	18	p.A289V (39%)	7
p.G719A (23%)^b^	18	p.I706T (22%)^b^	18
p.G719A (44%)^b^	18	p.E709A (44%)^b^	18
p.G719A (26%)^b^	18	p.E709K (25%)^b^	18
p.G719C (61%)^b^	18	p.E709A (60%)^b^	18
p.G719A (36%)	18	p.S768I (46%)	20
p.G719A (63%)	18	p.G779C (77%)	20
p.G719A (40%)	18	p.S768I (46%)	20
p.G719A (19%)	18	p.T790M (16%)	20
p.G719C (77%)	18	p.S768I (76%)	20
p.G719C (41%)	18	p.S768I (42%)	20
p.G719S (11%)	18	p.S768I (10%)	20
p.G719S (46%)	18	p.S768I (42%)	20
p.G719A (60%)	18	p.L861Q (66%)	21
p.G719D (21%)	18	p.L861Q (19%)	21
p.G719S (31%)	18	p.L861Q (25%)	21
p.L747_T751del (30%)^b,c^	19	p.K754Q (30%)^b,c^	19
p.L747_P753delinsS (12%)	19	p.T790M (7.3%)	20
p.L858R (16%)	21	p.R108K (21%)	3
p.L858R (14%)	21	p.R108K (21%)	3
p.L858R (15%)	21	p.A289V (18%)	7
p.L861Q (75%)	21	p.A289T (78%)	7
p.L858R (10%)	21	p.E709K (13%)	18
p.L858R (29%)	21	p.I744M (36%)	19
p.L858R (7.6%)	21	p.T790M (65%)	20
p.L858R (20%)	21	p.S768I (24%)	20
p.L861Q (17%)	21	p.T790M (19%)	20

^a^Percentage in the parenthesis indicates variant allele frequency.

^b^Located within the same allele.

^c^Double *EGFR* mutations seen in this tumor may also be categorized as a complex exon 19 deletion (c.2238_2260delinsATCTCCGC, p.L747_K754delinsSPQ).

### Concomitant *EGFR* and *PIK3CA* mutations

There were 7 tumors with both *EGFR* and *PIK3CA* mutations. In contrast to double *EGFR* mutations, *EGFR* VAFs were not concordant with those of the coexisting *PIK3CA* mutations (Figure 2B, *r* = 0.07). VAFs of the *EGFR* mutations were equivalent to or higher than those of the coexisting *PIK3CA* mutations (*P* = 0.009 by paired Student’s *t*-test).

### Concomitant *BRAF* and *KRAS* mutations

Activating *KRAS* mutation was detected in 11 (18%) of 63 *BRAF*-mutated tumors, including one (3.0%) of 33 tumors with a kinase-activated *BRAF* mutation, 5 (31%) of 16 with a kinase-impaired *BRAF* mutation, and 5 (36%) of 14 with a *BRAF* mutation of unknown kinase activity (Table 5) [19–22]. None of the tumors with concomitant *BRAF* and *KRAS* mutations had the p.V600E mutation. The incidence of concomitant *BRAF* mutations with activating *KRAS* mutations is significantly lower in tumors with kinase-activated mutants than those with kinase-impaired (*P* = 0.01) or kinase-unknown mutants (*P* = 0.006).

**Table 5:** Concomitant *BRAF* mutation and activating *KRAS* mutation in lung cancers

mut 1 (VAF%)	mut 2 (VAF%)	BRAF kinase activity
*KRAS* p.Q61H (12%)	*BRAF* p.G464V (9.5%)	activated^a^ [19]
*KRAS* p.G12D (19%)	*BRAF* p.G466E (3.3%)	Impaired [19]
*KRAS* p.A146T (30%)	*BRAF* p.G466V (35%)	Impaired [19]
*KRAS* p.G12D (19%)	*BRAF* p.G466V (14%)	Impaired [19]
*KRAS* p.G12V (14%)	*BRAF* p.D594G (2.6%)	Impaired [20]
*KRAS* p.Q61H (24%)^b^	*BRAF* p.D594N (5.7%)^b^	Impaired [21]
*KRAS* p.G12V (16%) ^b^	*BRAF* p.T440I (13%)^b^	unknown
*KRAS* p.G12C (14%)	*BRAF* p.G466L (12%)	unknown
*KRAS* p.G12R (12%)^b^	*BRAF* p.G469R (11%)^b^	unknown
*KRAS* p.G13D (25%)^b^	*BRAF* p.G469V (20%)^b^	unknown
*KRAS* p. A146T (11%)	*BRAF* p.K601N (11%)	unknown

VAF: variant allele frequency.

^a^Categorized as intermediate activity mutants by Wan et al. [19].

^b^These 4 cases have been reported previously [22].

## DISCUSSION

NGS has been clinically validated for mutational profiling of lung cancer [7, 23, 24]. We have previously shown a test feasibility of 94% among the first 625 lung cancer specimens submitted for NGS testing. This included an approximately 3% of specimens rejected due to inadequate specimens and approximately 3% of specimens failed the NGS assay [14]. This retrospective analysis of 1006 lung cancers reaffirms the strength of NGS in clinical mutational profiling. NGS demonstrates a great analytic sensitivity, broad reportable ranges, and accurate detection and annotation of complex indel mutations. With an analytic sensitivity of 10–20% VAF, Sanger sequencing would have missed 7.8% or 27% of *EGFR* mutations with less than 10% or 20% VAFs in this series. The analytic sensitivity can be improved to approximately 1–5% VAF by mutation-specific real time PCR assays such as cobas EGFR mutation test and therascreen EGFR RGQ PCR Kit, which were designed to detect only hot spot *EGFR* mutations [9–13].

NGS detected a variety of uncommon mutations located outside the reportable ranges of cobas and therascreen tests, including 4 codon 108 mutations and 4 codon 289 mutations involving the extracellular domain of EGFR. These mutations are more prevalent in glioblastomas and their clinical significance in lung cancer is not known [25]. NGS also detected 3 complex exon 19 mutations not included in the design of cobas and therascreen tests and additional 3 not included in the therascreen test. Among 18 tumors with TKI-resistant exon 20 insertion mutations detected by NGS, cobas test would have detected only 8 with c.2300_2308dup or c.2317_2319dup, and therascreen test would have detected only 3 with c.2317_2319dup. Both cobas and therascreen tests would also have missed TKI-sensitive exon 20 insertion mutations, such as p.A763_Y764insFQEA, which can be detected by this NGS assay as shown previously [26, 27].

Complex exon 19 deletion mutations may not be accurately characterized by Sanger sequencing [28], partly because the accompanied single nucleotide change or second deletion change may be difficult to interpret in the presence of an underlying deletion mutation, especially at a lower level of VAF. Furthermore, Sanger sequencing cannot distinguish if the two adjacent sequence changes are located within the same allele or different alleles without laborious cloning of the PCR amplicons for sequencing. NGS platforms provide individual sequencing information from a single molecule and, therefore, can confirm that the two sequence changes are always located within the same allele to form a complex exon 19 deletion. With accurate detection and annotation of the complex exon 19 deletion, further studies are warranted to elucidate if the point mutation component of the complex exon 19 deletion mutations may decrease TKI efficacy.

Application of assays with broader reportable ranges may shed light on the significance of uncommon mutations. For example, mutations involving codon 33 of the *KRAS* gene were recently found to be oncogenic [29]. NGS detected 17 unique *PIK3CA* mutations including 3 novel ones. Commonly reported mutations involving codons 542, 545 and 1047 accounted for only 65% of the *PIK3CA* mutations. A total of 24 unique *BRAF* mutations including 4 novel ones were detected among 63 *BRAF*-mutated lung cancers. The p.V600E mutant accounted for only 24% of *BRAF* mutations while kinase-impaired *BRAF* mutants involving codons 466 and 594 were seen in 25% of *BRAF* mutations. In a previous study of combined lung cancer, colorectal cancer and melanoma specimens, kinase-impaired *BRAF* mutants were associated with a higher incidence of a concomitant activating *KRAS*/*NRAS* mutation [22]. This is confirmed by a larger cohort of lung cancer specimens in this study. *In vitro* studies have shown that in the presence of oncogenic RAS proteins, kinase-impaired BRAF forms a complex with CRAF and leads to hyperactivation of the CRAF/MEK/ERK cascade, suggesting MEK inhibitors or CRAF inhibitors may benefit patients with concomitant kinase-impaired *BRAF* mutation and activating *RAS* mutation [20, 21]. Dabrafenib (a selective BRAF inhibitor) alone or combined with trametinib (a MEK inhibitor) has shown efficacy in p.V600E-mutated lung cancers [30, 31]. The NCI-Molecular Analysis for Therapy Choice (NCI-MATCH) Trial also includes an arm for targeting tumors with non-p.V600E *BRAF* mutations with trametinib (https://www.cancer.gov/about-cancer/treatment/clinical-trials/nci-supported/nci-match, accessed 1/19/2017).

False negative results were a major concern when NGS platforms were initially implemented in the clinical diagnostic setting. During our clinical validation of this NGS platform, we have found that Torrent Variant Caller alone may miss *EGFR* p.L858R [18]. Therefore, we have included IGV inspection of each amplicon as a second analysis pipeline. We also examined the indel mutations within *EGFR* exons 19 and 20 by a sizing assay. All *EGFR* mutations, missed by older versions of Torrent Variant Caller, were observed by IGV. Although recent versions did not miss mutations in lung cancers, version 5.0.2.1 (since December 2015) did miss a 8.4% *KIT* p.K558_D572del (45-base deletion mutation) in a gastrointestinal stromal tumor specimen and version 5.0.4.0 (since September 2016) missed a 2.6% *BRAF* p.V600E in a melanoma specimen with less than 10% estimated tumor cellularity (unpublished data). All exons 19 and 20 indel mutations detected by the sizing assay were also observed by IGV inspection. The results indicate that indel mutations of 18 or less bases can be reliably detected by Torrent Suite analysis combined with direct visual inspection of the binary sequence alignment/map file using IGV. Longer indel mutations such as internal tandem duplication mutations of the *FLT3* gene may not be detected by NGS assays without bioinformatics pipelines designed for longer indel mutations [32, 33].

Consistent VAFs over a 4-year period in positive control specimens highlighted the precise quantitative nature of NGS assays [27, 34]. Analysis of VAF may yield important information regarding mutant allele-specific imbalance (such as gene amplification or loss of heterozygosity), tumor heterogeneity, and germline mutations [7, 34, 35]. We have shown that lower than expected VAF indicated tumor heterogeneity while higher than expected VAF indicated mutant allele-specific imbalance [34, 35]. In this study, we found an equivalent higher *EGFR* VAF than the coexisting *PIK3CA* VAF. The results suggest a higher incidence of mutant allele-specific imbalance (most likely duplication or amplification) of the *EGFR* gene or the presence of *PIK3CA* mutation in a subpopulation of the *EGFR*-mutated tumors which may contribute to TKI resistance [27]. This was confirmed by subarea analysis of a specimen containing 2.8% *PIK3CA* mutation and 12% *EGFR* p.L858R mutation. Only one of 8 fragments showed both mutations with concordant VAFs (data not shown). In contrast, VAFs of the doublet *EGFR* mutations were highly concordant except for one specimen with 7.6% p.L858R and 65% p.T790M in a context of 11–30% estimated tumor cellularity, suggesting a germ-line p.T790M mutation [36].

Doublet *EGFR* mutations are not uncommon (1.6% to 14%) [37–40]. A summary from 66 publications showed 96 (6%) doublets in 1621 *EGFR*-mutated lung cancers [41], including several tumors which were indeed complex exon 19 deletion. The incidence of doublet *EGFR* mutations is higher by using sensitive assays with broader reportable ranges such as NGS assays. We found two *EGFR* mutations in 29 (16%) of 187 *EGFR*-mutated tumors while 11 tumors with complex exon 19 deletion were excluded. Concordant VAFs between two mutations suggests one dominant tumor population with two mutations rather than two tumor populations each containing a mutation. Both mutations may be needed to initiate the founder clone or an *EGFR*-mutated subclone that has become the dominant tumor population after acquiring the second mutation. Concomitant mutations between exon 19 deletion, exon 20 insertion and p.L858R are uncommon [41]. Only one doublet consisting of exon 19 deletion and p.L858R was reported among 1621 *EGFR*-mutated lung cancers [41]. In contrast, non-p.L858R missense mutations were often seen in doublet mutations, suggesting a lower oncogenic potential of these mutations. *In vitro* studies have shown a comparable or higher level of catalytic phosphorylating activity in mutants involving conservative codons at 709, 719, 768, 790 and 861 compared to the wild-type, but a lower level of kinase activity with respect to exon 19 deletion or p.L858R mutants [42–45]. Significant enhancement of kinase activity observed in doublet with p.T790M and p.L858R or exon 19 deletion suggests additive oncogenic effect from p.T790M [43, 44].

NGS demonstrates a high analytic sensitivity, quantitative measurement of VAF, single molecule sequencing of complex exon 19 deletion, and broad reportable ranges with simultaneous detection of doublet *EGFR* mutations and concomitant *BRAF* and *KRAS* mutations in the clinical diagnostic setting. Further studies are warranted to elucidate the clinical and/or biological significance of uncommon mutations, doublet non-p.L858R missense mutations of *EGFR*, and concomitant kinase-impaired *BRAF* mutations in lung cancers.

## MATERIALS AND METHODS

### Materials

NGS was conducted in 1103 formalin-fixed paraffin-embedded (FFPE) specimens with a diagnosis of adenocarcinoma *in situ* (5 specimens), invasive adenocarcinoma (1033 specimens), adenosquamous carcinoma (10 specimens) or non-small cell carcinoma (55 specimens) of lung submitted to the Molecular Diagnostics Laboratory at The Johns Hopkins Hospital between April 2013 and June 2016. Specimens with prior TKI therapy were not included. There were 499 (45%) resection specimens, 341 (31%) biopsy specimens, and 204 (19%) fine needle aspiration specimens, 55 (5.0%) pleural or pericardial effusion specimen, 2 bronchial brushing specimen, one bronchoalveolar lavage specimen and one curetting of bone (Supplementary Table 6). Twenty-nine (2.6%) specimens failed (Supplementary Table 6). The remaining 1074 specimens with successful NGS were submitted from 1006 tumors of 987 patients (Supplementary Table 7). Tissue blocks with adequate tumor cellularity were selected by the pathologists who made the diagnosis. One hematoxylin & eosin (H&E) slide followed by 5–10 unstained slides and one additional H&E slide were prepared with PCR precautions. The H&E slide was examined and marked by PI (pulmonary pathologist), MTL and/or GZ (molecular pathologists) for subsequent macro-dissection of FFPE neoplastic tissues from 3–10 unstained slides of 5 or 10-micron thick sections. DNA was isolated from the designated area(s) using the Pinpoint DNA Isolation System (Zymo Research, Irvine, CA), followed by further purification via the QIAamp Mini Kit (Qiagen, Valencia, CA) [46]. DNA concentration was measured by Qubit 2.0 Fluorometer (Life Technologies, Carlsbad, California). Specimens with concentration less than 10 ng/µl or bony specimens were concentrated with Amicon Ultra 0.5 ml centrifugal filters with ultracel-30K membrane (Millipore Sigma, Darmstadt, Germany) after April 2015. The Johns Hopkins Medicine institutional review board granted approval to this study.

### Next generation sequencing (NGS)

NGS was conducted using AmpliSeq Cancer Hotspot Panel (v2) for targeted multi-gene amplification, as described previously [18, 34]. Briefly, we used the Ion AmpliSeq Library Kit 2.0 for library preparation, Ion OneTouch 200 Template Kit v2 DL (replaced by Ion Personal Genome Machine Hi-Q OT2 Kit lately) and Ion OneTouch 2 Instrument for emulsion PCR and template preparation, and the Ion Personal Genome Machine 200 Sequencing Kit (replaced by Ion Personal Genome Machine Hi-Q Sequencing Kit lately) with the Ion 318 Chip and Personal Genome Machine as the sequencing platform (Life Technologies). The DNA input ranged from 1 ng to 30 ng, as measured by Qubit 20 Fluorometer (Life Technologies). Up to 8 specimens were barcoded using Ion Xpress Barcode Adapters (Life Technologies) for each Ion 318 chip. One to three controls (a non-template control, a normal peripheral blood control from a male, and/or positive control specimens) were included in each chip. Positive controls were mixed DNA specimens from several cell lines with known mutations as reported previously [27, 34].

Redundant bioinformatics pipelines are essential for NGS analysis, as a single analysis pipeline may give false negative and false positive results. Direct visual inspection of the binary sequence alignment/map file using the Broad Institute’s Integrative Genomics Viewer (IGV) (http://www.broadinstitute.org/igv/) was implemented during the validation processes of this assay after we found that Torrent Variant Caller missed the most common *EGFR* point mutation (p.L858R) in a lung cancer specimen [18, 47]. In this study, sequencing data were analyzed using Torrent Suite (Life Technologies) as described previously [18]. Mutations were identified and annotated through both Torrent Variant Caller (Life Technologies) and direct visual inspection of the binary sequence alignment/map file using IGV. All specimens were analyzed for *AKT1* (NM_005163), *BRAF* (NM_004333), *EGFR* (NM_005228), *ERBB2* (NM_004448), *KRAS* (NM_033360), *NRAS* (NM_002524) and *PIK3CA* (NM_006218) genes. During our validation of this NGS assay, a cutoff of background noise at 2% was chosen for single nucleotide variant according to a study of 16 non-neoplastic FFPE tissues [18]. We also developed a statistical model to determine the read depth needed for a given percent tumor cellularity and number of functional genomes. With sufficient DNA input, the limit of detection is dictated by the depth of coverage (or number of sequencing reads). Approximately 150 and 500 reads is needed to detect a heterozygous mutation at a 99% confidence in a specimen with 20% and 10% tumor cellularity, respectively. The reportable ranges and reference ranges for the 7 genes have been reported previously [27, 34]. *BRAF* mutation data before September 2014 has also been published previously together with colorectal cancer specimens and melanoma specimens [22].

### Alternative assays

Insertion/deletion mutations within exons 19 and 20 of the *EGFR* genes were also examined by sizing using capillary electrophoresis. PCR followed by capillary electrophoresis was conducted as described previously [18]. This was discontinued after April 2014. Mutations not reported in the COSMIC database were confirmed by either Sanger sequencing or pyrosequencing. This policy was also discontinued after April 2015.

### Statistical analysis

Student’s *t*-test, χ^2^ test or Fisher exact test was performed to calculate *P* values. Correlation of frequencies between two mutations was examined by Spearman’s rank correlation coefficient (denoted as *r*) using the GraphPad Prism software (GraphPad Software, ver5, La Jolla, CA) as described previously [48].

## SUPPLEMENTARY MATERIALS FIGURES AND TABLES




