# Supplementary material for: Clinical mutational profiling of 1006 lung cancers by next generation sequencing
Source: Oncotarget. 2017 May 20;8(57):96684–96. doi: 10.18632/oncotarget.18042 (PMC5722514; doi:10.18632/oncotarget.18042)
Supplement: Supplementary file 1 [file oncotarget-08-96684-s001.pdf]

Clinical mutational profiling of 1006 lung cancers by next generation sequencing

Supplementary Materials

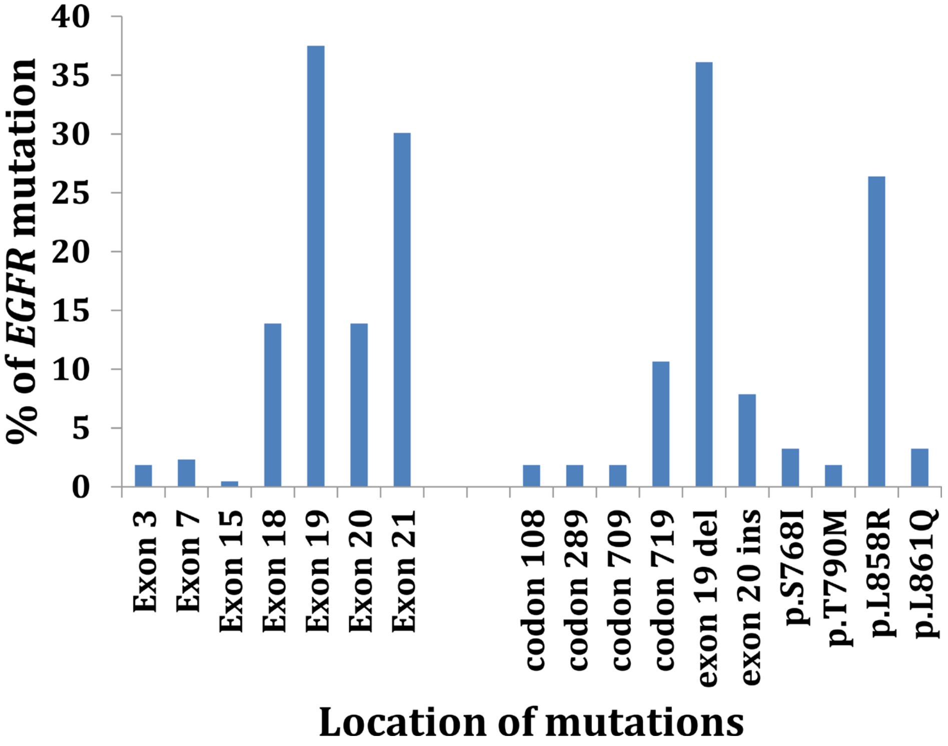

Supplementary Figure 1: Distribution of 216 *EGFR* mutations. The mutations were located within exon 18 in 30 (14%) of 216 *EGFR* mutations including 11% codon 719 mutations, 38% exon 19 mutations (36% exon 19 deletion mutations), 14% exon 20 mutations (7.9% exon 20 insertion mutations), and 30% exon 21 mutations (26% p.L858R).

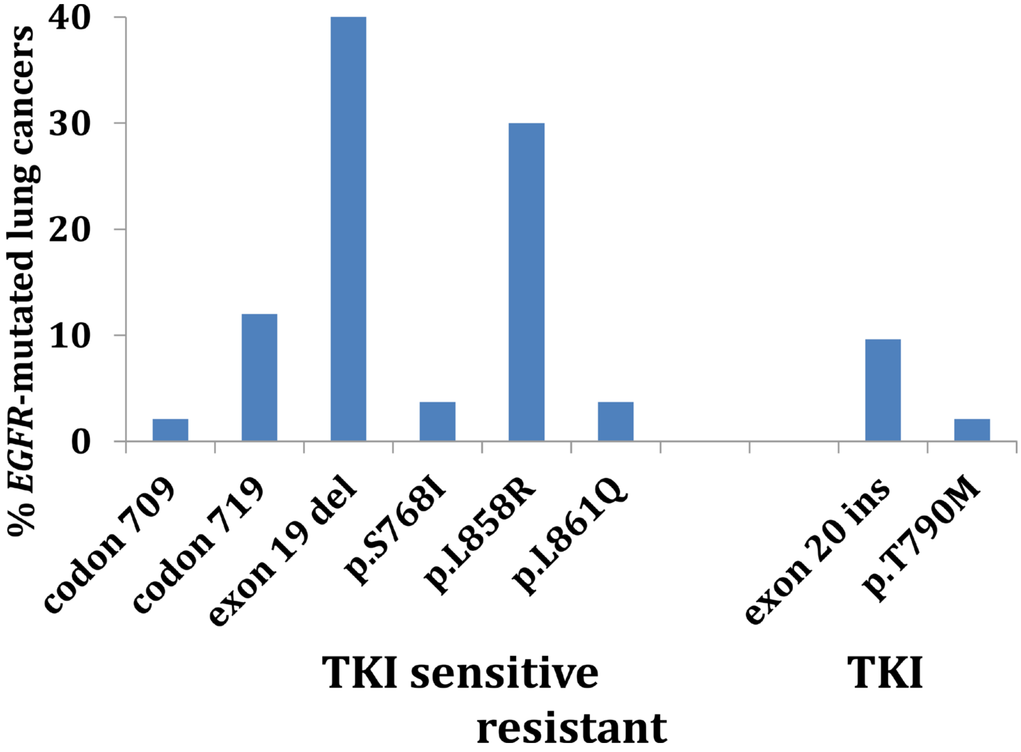

Supplementary Figure 2: Tyrosine kinase inhibitor (TKI) sensitive or resistant mutations among 187 *EGFR*-mutated lung cancers. The common TKI-sensitive mutations included exon 19 deletion mutations (42% of 187 *EGFR*-mutated tumors), p.L858R (30%), codon 719 mutations (12%), p.S768I (3.7%), p.L861Q (3.7%) and codon 709 mutations (2.1%). The TKI-resistant mutations within exon 20 included insertion mutations (9.6% of 187 *EGFR*-mutated tumors) and p.T790M (2.1%).

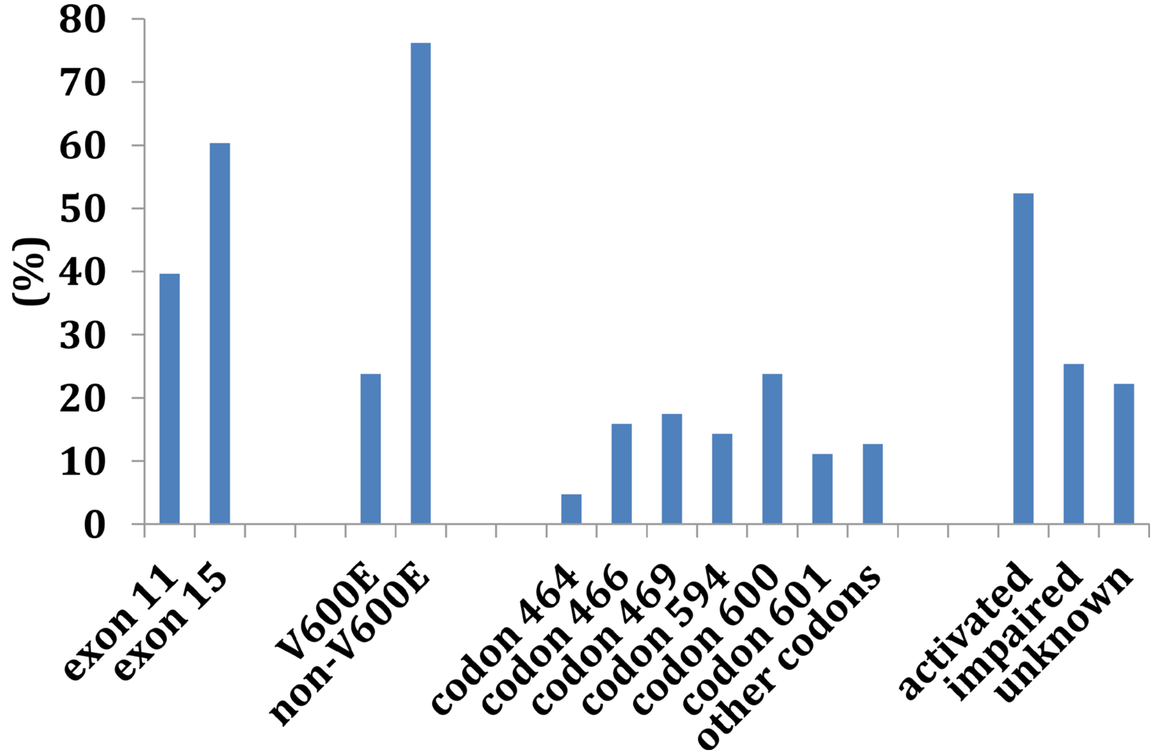

Supplementary Figure 3: Distribution of *BRAF* mutations according to location and BRAF kinase activity.

Supplementary Table 1: Mutant allele frequency of positive control specimens[a]

Mutations	Lung cancel panel control (n = 43)[b]
AKT1 p.E17K	7.2 ± 1.0%
AKT1 p.E49K	7.1 ± 1.0%
BRAF p.V600E	12.1 ± 1.0%
EGFR p.T790M	9.4 ± 1.3%
EGFR p.L858R	6.1 ± 1.0%
ERBB2 p.G776V	8.4 ± 0.8%
KRAS p.G12C	21.8 ± 1.2%
KRAS p.P121H	7.8 ± 0.8%
NRAS p.Q61R	9.1 ± 0.8%
PIK3CA p.H111E	6.7 ± 1.1%
PIK3CA p.H1047R	14.4 ± 1.1%

[a]Only results between July 2015 and June 2016 are included. Results before July 2015 have been reported previously [27, 34].
[b]Mean ± standard deviation.

Supplementary Table 2: *EGFR* mutations in 1006 lung cancers. See Supplementary_Table_2

Supplementary Table 3: *KRAS* and *NRAS* mutations in 1006 lung cancers

Gene	cDNA change	Amino acid change	Exon	Number of case
KRAS				
	c.15A>T	p.K5N	2	1
	c.22G>A	p.V8I[c]	2	1
	c.35G>C	p.G12A	2	24
	c.34G>T	p.G12C	2	124
	c.35G>A	p.G12D	2	41
	c.34_35delinsTT	p.G12F	2	8
	c.34G>C	p.G12R	2	4
	c.34G>A	p.G12S	2	7
	c.35G>T	p.G12V	2	81
	c.37G>T	p.G13C	2	13
	c.38G>A	p.G13D	2	20
	c.97G>C	p.D33H[c]	2	1
	c.97G>T	p.D33Y[c]	2	1
	c.127C>G	p.Q43E[c]	3	1
	c.183A>C or T[a]	p.Q61H	3	25
	c.182A>T[b]	p.Q61L	3	6
	c.182A>G	p.Q61R	3	1
	c.436G>A	p.A146T	4	3
	c.437C>T	p.A146V	4	3
NRAS				
	c.34G>T	p.G12C	2	2
	c.35G>A	p.G12D	2	4
	c.143G>C	p.G48A[c]	3	1
	c.183A>T	p.Q61H	3	1
	c.182A>T	p.Q61L	3	2
	c.182A>G	p.Q61R	3	1

[a]10 with c.183A>C and 15 with c.183A>T.
[b]One with c.182_183delinsTG and 5 with c.182A>T.
[c]Not reported in the COSMIC database.

Supplementary Table 4: *BRAF* mutations in 1006 lung cancers

cDNA change	Amino acid change	Exon	Number of case[a]	kinase activity[b]
c.1391G>T	p.G464V	11	3 (2)	activated[b]
c.1397G>C	p.G466A	11	2 (2)	activated[b]
c.1406G>C	p.G469A	11	6 (1)	activated[b]
c.1785T>A	p.F595L	15	1	activated[b]
c.1799T>A	p.V600E	15	15 (7)	activated
c.1801A>G	p.K601E	15	6 (3)	activated
c.1397G>A	p.G466E	11	1	impaired
c.1396G>C	p.G466R	11	1 (1)	impaired
c.1397G>T	p.G466V	11	5 (1)	impaired
c.1781A>G	p.D594G	15	5 (4)	impaired
c.1780G>C	p.D594H	15	1 (1)	impaired
c.1780G>A	p.D594N	15	3 (2)	impaired
c.1319C>T	p.T440I[c]	11	1 (1)	unknown
c.1396_1397delinsTT	p.G466L[c]	11	1	unknown
c.1405G>A	p.G469R	11	1 (1)	unknown
c.1405_1406delinsTC	p.G469S	11	1 (1)	unknown
c.1406G>T	p.G469V	11	3 (1)	unknown
c.1762C>T	p.L588F	15	1 (1)	unknown
c.1786G>T	p.G596C	15	1	unknown
c.1786G>C	p.G596R	15	1 (1)	unknown
c.1799_1801del	p.V600_K601delinE	15	1 (1)	unknown
c.1803A>T	p.K601N	15	1	unknown
c.1825_1826delinsTT	p.Q609L[c]	15	1 (1)	unknown
c.1831G>C	p.E611Q[c]	15	1 (1)	unknown

[a]Number in the parentheses indicates the number of *BRAF*-mutated lung cancers detected before September 2014 [22].
[b]Categorized as intermediate activity mutants by Wan et al. [19].
[c]Not reported in the COSMIC database.

Supplementary Table 5: *AKT1, PIK3CA and ERBB2* mutations in 1006 lung cancers

Gene	cDNA change	Amino acid change	Exon	Number of case
AKT1				
	c.49G>A	p.E17K	3	3
	c.74G>A	p.R25H[a]	3	1
PIK3CA				
	c.263G>A	p.R88Q	1	1
	c.1028A>T	p.Y343F	4	1
	c.1031T>G	p.V344G	4	1
	c.1034A>T	p.N345I	4	1
	c.1048G>A	p.D350N	4	1
	c.1193G>T	p.R398L[a]	6	1
	c.1201C>T	p.R401*[a]	6	1
	c.1348C>G	p.H450D[a]	7	1
	c.1624G>A	p.E542K	9	6
	c.1357G>A	p.E453K	9	1
	c.1634A>C	p.E545A	9	1
	c.1633G>A	p.E545K	9	11
	c.1637A>T	p.Q546L	9	1
	c.1637A>C	p.Q546P	9	1
	c.3140A>G	p.H1047R	20	6
	c.3143A>G	p.H1048R	20	1
	c.3145G>C	p.G1049R	20	1
ERBB2				
	c.2270A>G	p.E757G[a]	19	1
	c.2313_2324dup	p.A771_M774dup	20	8
	c.2326_2327insTGT	p.G776delinsVC	20	1
	c.2327delinsTCGT	p.G776delinsVV[a]	20	1
	c.2331-2339dup	p.G778_P780dup	20	2

[a]Not reported in the COSMIC database.

Supplementary Table 6: 1103 specimens with lung cancers

	Total	Failed NGS[a]
Resection/excison biopsy	499	4 (0.8%)
lung	361	1
brain	62	0
pleura	32	0
lymph node	24	0
bone	6	3
liver	3	0
pericardium	3	0
others	8	0
Core biopsy	341	20 (5.9%)
lung	251	12
lymph node	37	1
liver	19	1
bone	12	3
adrenal gland	4	1
others	18	2
Fine needle aspiration	204	3 (1.5%)
lymph node	140	1
lung	57	1
bone	3	1
others	4	0
Effusion	55	0 (0%)
pleural effusion	50	0
pericardial effusion	5	0
Bronchial brushing	2	0
Bronchoalveolar lavage	1	1
Curettings of bone	1	1

[a]Percentage in the parentheses indicates the failure rate.

Supplementary Table 7: 1074 specimens from 1006 tumors of 987 patients with lung cancers

	Patient (*n*)	Tumor (*n*)
Single specimen	906	906
Paired specimens	75	92
Same tumor[a]	16	16
Different metastatic site	10	10
Primary and metastatic site	22	25
same mutation	19	19
different mutation	3	6
Different lung nodules	27	41
same mutation	13	13
different mutation	14	28
Three specimens[b]	6	8
Total	987	1006

[a]Biopsy, fine needle aspiration or resection of the same tumor.
[b]One patient with 3 lung nodules of different mutation status.
