# Supplementary material for: Clinical mutational profiling of 1006 lung cancers by next generation sequencing
Source: Oncotarget. 2017 May 20;8(57):96684–96. doi: 10.18632/oncotarget.18042 (PMC5722514; doi:10.18632/oncotarget.18042)
Supplement: Supplementary file 2 [file oncotarget-08-96684-s002.docx]

**Supplementary Table 2: *EGFR* mutations in 1006 lung cancers.**

| cDNA change | Amino acid change | Exon | Number of case |
| --- | --- | --- | --- |
| c.322A>G | p.R108G | 3 | 1 |
| c.323G>A | p.R108K | 3 | 3 |
| c.844G>A | p.E282K^c^ | 7 | 1 |
| c.865G>A | p.A289T | 7 | 1 |
| c.866C>T | p.A289V | 7 | 3 |
| c.1751A>G | p.H584R^c^ | 15 | 1 |
| c.2117T>C | p.I706T | 18 | 1 |
| c.2126A>C | p.E709A | 18 | 2 |
| c.2125G>A | p.E709K | 18 | 2 |
| c.2127_2129del | p.E709_T710delinsD | 18 | 1 |
| c.2156G>C | p.G719A | 18 | 13 |
| c.2155G>T | p.G719C | 18 | 5 |
| [c.2156G>A](http://cancer.sanger.ac.uk/cosmic/mutation/overview?id=18425) | p.G719D | 18 | 1 |
| c.2155G>A | p.G719S | 18 | 3 |
| c.2171G>C | p.G724A^c^ | 18 | 1 |
| c.2170G>A | p.G724S | 18 | 1 |
| c.2189T>G | p.L730R^c^ | 19 | 1 |
| c.2232C>G | p.I744M | 19 | 1 |
| c.2235_2249del or c.2236_2250del^a^ | p.E746_A750del | 19 | 51 |
| c.2236_2248delinsGCAC | p.E746_A750delinsAP | 19 | 1 |
| c.2235_2251delinsAATTC | p.E746_T751delinsIP | 19 | 1 |
| c.2237_2252delinsT | p.E746_T751delinsV | 19 | 1 |
| c.2237_2255delinsT | p.E746_S752delinsV | 19 | 2 |
| c.2237_2257delinsTCT | p.E746_P753delinsVS | 19 | 1 |
| c.2239_2248delinsC or c.2238_2248delinsGC^b^ | p.L747_A750delinsP | 19 | 4 |
| c.2240_2254del | p.L747_T751del | 19 | 3 |
| c.2239_2251delinsC | p.L747_T751delinsP | 19 | 2 |
| c.2239_2252delinsCA | p.L747_T751delinsQ | 19 | 1 |
| c.2239_2256del | p.L747_S752del | 19 | 2 |
| c.2239_2258delinsCA | p.L747_P753delinsQ | 19 | 1 |
| c.2240_2257del | p.L747_P753delinsS | 19 | 7 |
| [c.2240_2264>CGAAAGG](http://cancer.sanger.ac.uk/cosmic/mutation/overview?id=1667026) | p.L747_A755delinsSKG | 19 | 1 |
| c.2260A>C | p.K754Q | 19 | 1 |
| c.2302_2303insTTGCCA | p.A767_S768insIA | 20 | 1 |
| c.2302_2303insCGCTGGCCA | p.A767_S768insTLA | 20 | 1 |
| c.2300_2308dup^d^ | p.A767_V769dup | 20 | 5 |
| c.2303G>T | p.S768I | 20 | 7 |
| c.2303G>T and c.2305G>T | p.S768_V769delinsIL | 20 | 1 |
| c.2303_2311dup | p.S768_D770dup | 20 | 1 |
| c.2308_2309insGTT | p.D770delinsGY | 20 | 1 |
| c.2310_2311insTAC | p.D770_N771insY | 20 | 1 |
| c.2311_2312delinsGGGTT | p.N771delinsGF | 20 | 1 |
| c.2311_2319dup | p.N771_H773dup | 20 | 3 |
| c.2317_2319dup^d,e^ | p.H773dup | 20 | 3 |
| c.2335G>T | p.G779C | 20 | 1 |
| c.2369C>T | p.T790M | 20 | 4 |
| c.2571Gdel | p.L858fs*44^c^ | 21 | 1 |
| c.2573T>G | p.L858R | 21 | 57 |
| c.2582T>A | p.L861Q | 21 | 7 |

^a^ 35 with c.2235_2249del and 16 with c.2236_2250del.

^b^ 3 with c.2239_2248delinsC and one with c.2238_2248delinsGC.

^c^ Not reported in the COSMIC database. p.E282K has been reported as a rare germ-line variant (rs199796955). In the context of 30-50% estimated tumor cellularity and 52% variant allele frequency, p.H584R may also represent a rare germline variant. The p.L858fs*45 mutation was confirmed by Sanger sequencing (data not shown).

^d^ Tumors with c.2300_2308dup (or 2307_2308insGCCAGCGTG) or c.2317_2319dup (or 2319_2320insCAC) can be detected by cobas EGFR test. Other exon 20 insertion/duplication mutations may not be detected by cobas EGFR test.

^e^ Tumors with c.2317_2319dup can be detected by therascreen EGFR test. Other exon 20 insertion/duplication mutations may not be detected by therascreen EGFR test.
